# Interaction of glutathione S-transferase polymorphisms and tobacco smoking during pregnancy in susceptibility to autism spectrum disorders

**DOI:** 10.1038/s41598-019-39885-w

**Published:** 2019-03-01

**Authors:** Vanja Mandic-Maravic, Vesna Coric, Marija Mitkovic-Voncina, Miroslav Djordjevic, Ana Savic-Radojevic, Marko Ercegovac, Marija Matic, Tatjana Simic, Dusica Lecic-Tosevski, Oliver Toskovic, Tatjana Pekmezovic, Marija Pljesa-Ercegovac, Milica Pejovic-Milovancevic

**Affiliations:** 1 Institute of Mental Health, Belgrade, Serbia; 2 Faculty of Medicine, University of Belgrade, Belgrade, Serbia; 3 Institute of Medical and Clinical Biochemistry, Belgrade, Serbia; 4 University Children’s Hospital, Belgrade, Serbia; 5 Neurology Clinic, Clinical Center of Serbia, Belgrade, Serbia; 6 Serbian Academy of Sciences and Arts, Belgrade, Serbia; 7 Faculty of Philosophy, Department of Psychology, Belgrade, Serbia; 8 Institute of Epidemiology, Belgrade, Serbia

## Abstract

Autism spectrum disorders (ASD) are a group of complex psychiatric disorders, with a proposed gene-environment interaction in their etiology. One mechanism that could explain both the genetic and environmental component is oxidative stress. The aim of our study was to investigate the potential role of common polymorphisms in genes for glutathione transferase A1, M1, T1 and P1 in susceptibility to ASD. We also aimed to explore the possible oxidative stress - specific gene-environment interaction, regarding GST polymorphisms, maternal smoking tobacco during pregnancy (TSDP) and the risk of ASD. This case-control study included 113 children with ASD and 114 age and sex-matched controls. The diagnosis was made based on ICD-10 criteria and verified by Autism Diagnostic Interview – Revised (ADI-R). We investigated *GSTA1*, *GSTM1*, *GSTP1* and *GSTT1* genotypes and explored their individual and combined effects in individuals with ASD. Individual effect of GST genotypes was shown for *GSTM1 active* genotype decreasing the risk of ASD (OR = 0.554, 95%CI: 0.313–0.983, p = 0.044), and for *GSTA1 CC* genotype, increasing susceptibility to ASD (OR = 4.132, 95%CI: 1.219–14.012, p = 0.023); the significance was lost when genotype-genotype interactions were added into the logistic regression model. The combination of *GSTM1 active* and *GSTT1 active* genotype decreased the risk of ASD (OR = 0.126, 95%CI: 0.029–0.547, p = 0.006), as well as combination of *GSTT1 active* and *GSTP1 llelle* (OR = 0.170, 95%CI: 0.029–0.992, p = 0.049). Increased risk of ASD was observed if combination of *GSTM1 active* and *GSTP1 llelle* was present (OR = 11.088, 95%CI: 1.745–70.456, p = 0.011). The effect of TSDP was not significant for the risk of ASD, neither individually, nor in interaction with specific GST genotypes. Specific combination of GST genotypes might be associated with susceptibility to ASD, while it appears that maternal smoking during pregnancy does not increase the risk of ASD.

## Introduction

Autism spectrum disorders (ASD) represent a group of disorders which include symptoms such as impairment of social interactions, impairment of communication (both verbal and nonverbal) and restricted interests and repetitive behavior^1^. Epidemiological analyses showed that the prevalence of ASD has increased for 23% in the past 10 years^2^. The rise in prevalence could be accounted for by the recent changes in registering and diagnosis of ASD^3^, but also by the mechanism of gene-environment interaction^4^. The phenotypic manifestations of ASD are largely heterogeneous, which might be explained by complex interaction of genetic susceptibility with different risk factors, leading to an individual path of developing ASD in each person. The data of previous studies show that the period of susceptibility to environmental factors in ASD, might be the prenatal and perinatal period of development^5^. There have been many studies focused on the aforementioned issue, thus some of the main potential prenatal and perinatal risk factors have been well defined^6–8^.

It is hypothesized that superfamily of glutathione transferases (GSTs), enzymes that are not only responsible for catalyzing detoxification reactions, but also are important as part of antioxidant defense system and in cellular signaling, might contribute to the development of ASD^9–11^.

The explanation for the assumption lies in the fact that GSTs or, more precisely, genetic polymorphism observed in almost all classes of GST superfamily, might increase the individual susceptibility to environmental factors associated with ASD^12^, and also to oxidative stress^13^.

Glutathione transferases, as part of gene-environment interaction in development of ASD, were mentioned a decade ago by Williams *et al*. (2007) who noted higher risk of ASD in children of women with *GSTP1 Ile/Val* genotype, further suggesting that risk factors during pregnancy might result in higher risk of ASD in later life^10^.

In recent years, significantly increased levels of lead and mercury, as well as a decrease of GST activity were noted in patients diagnosed with ASD, when compared to typically developed controls^14^. Moreover, another study has shown a possible interaction between *GSTP1* polymorphism (*GSTP1 Ile/Ile* genotype) and the effect of blood manganese concentration^11^, but also between GST polymorphisms and exposure to aluminum^15^. Available data on other *GST* genotypes are scarce, still *GSTM1 null* and *GSTT1 null* genotypes, alone or in combination with *GSTP1* polymorphism, have been associated with risk of ASD^16–18^. Interestingly, *GSTA1* genotype has not been evaluated in terms of ASD susceptibility as yet.

Multiple lines of evidence which suggested that oxidative stress could represent the basis for the observed association between genetic, immunological and environmental factors underlying autism^13,19–21^, also suggested the role of glutathione transferases in ASD development, in particular when taking into account the important antioxidant role of GSTs^11,17,22^.

One of the explored prenatal risk factors in recent studies is smoking tobacco during pregnancy. Tobacco smoking during pregnancy (TSDP) has been associated with numerous adverse events, such as preterm birth and low birth weight^23^, factors known to be also associated with ASD^6,8,24,25^. TSDP has also been associated with epigenetic changes in the offspring, which persist well into adulthood^26^. Moreover, a study by Hultman *et al*. (2002) confirmed that TSDP might indeed be a risk factor associated with ASD^27^. Furthermore, multiple studies confirmed the association between “second hand” smoking and ASD^8,28^. The aforementioned findings were somewhat controversial as a large study by Maimburg&Vaeth (2006) didn’t show evidence that TSDP increases the risk for ASD^29^. What is more, a meta-analysis done in 2015^30^ also showed no association between maternal TSDP and risk for ASD. This meta-analysis included 15 cohort and case-control studies, and involved only those that examined active TSDP. The authors argued, despite the fact that several plausible hypotheses might explain the association between maternal smoking and ASD, and it would be expected to be significant, the results were negative^30^. A recent large population study done in Sweden has shown association of TSDP and severe mental disorders (bipolar disorder and schizophrenia), but further analysis showed that this association weakens significantly after using the family based approach to estimate the risk. The authors point to a possible influence of a hidden familial confounding factor, such as passive gene-environment interaction^31^. One of the possible explanation for conflicting results in studies focused on association between TSDP and ASD could be clarified by the individual susceptibility to those factors. The susceptibility might have genetic variation as an underlying mechanism. Namely, genetic polymorphism with consequential lack or change in the activity of enzymes involved in detoxification of tobacco smoke, a rich source of free radicals and numerous carcinogens, might represent the determinant of this individual susceptibility. In this line, GSTA1, GSTM1 and GSTP1 participate in conjugation of tobacco smoke metabolites with glutathione, thus enhancing their excretion in urine, while due to strong peroxidase activity they are among key components in cellular defense against free radical damage^9,32,33^.

Another environmental factor which can be associated with neurotoxicity and adverse effects on the fetus *in utero* is air pollution, known to contain various airborne toxicants and contaminants capable of inducing oxidative stress and mitochondrial damage *in vitro*^34,35^. Similarly to tobacco smoke, genetic variations influence individual capability for protection from air pollution.

Considering the fact that identifying new specific gene-environment interactions, as well as, elucidating in which way antioxidants contribute to redox imbalance in autism^13^ might help resolve the complex etiology of ASD, we aimed to investigate the association between common polymorphisms in genes encoding cytosolic glutathione transferase A1, M1, T1 and P1 and ASD. We also aimed to explore the possible oxidative stress - specific gene-environment interaction, regarding GST polymorphisms and TSDP and the risk of ASD.

## Materials and Methods

### Study population

The study was performed as a case-control study. The case group involved 113 ASD patients (92 males, 21 females, 9.36 ± 5.88 years old), included as consecutive referrals and treated as outpatients and inpatients at the Institute of Mental Health, Belgrade, Serbia. The inclusion criterion for the case group was the presence of any of the ASD. The diagnosis was verified by the ICD-10 criteria^36^, confirmed by a child psychiatrist with experience in diagnosing ASD. The evaluation was done through clinical interview with a parent and examination of a child. Besides clinical interview and criteria, the diagnosis was verified by the Autism Diagnostic Interview – Revised (ADI-R)^37^, conducted by a trained child psychiatrist.

The control group consisted of 114 age and sex group-matched controls, recruited from the Urology and Orthopedic Department of University Children’s Hospital, Belgrade, Serbia. Control subjects suffered from unintentional injuries (fractures) and urogenital tract disorders (phimosis, chryptorchismus, penal curvature), and were selected consecutively, at the same time at which cases were collected. The exclusion criterion for controls was personal or family history of neurological or psychiatric disorder, as well as any kind of developmental delays. The difference in age and sex distribution within the group level was not statistically significant.

### Instruments

#### *Autism Diagnostic Interview - Revised *

*(ADI-R*)^37^. ADI-R is a standardized semi structured parent/caregiver interview, created for the assessment of signs of ASD. It comprises 93 items, and evaluates child’s early development, development of language, functioning of language and communication, loss of speech and motor skills, social development and play, interests and behavior. The description for each item, given by the parent/caregiver is made for childhood (ever) and current behavior. Specific items describing social reciprocity, communication and restricted, repetitive and stereotyped behavior (RRSB) are used to reach the scores for these three domains (ADI-R A, ADI-R B and ADI-R C score, respectively). Higher scores account for greater impairment – worse symptoms. In this study, the interview was administered by certified child psychiatrists.

#### Sociodemographic and exposure questionnaire

The questionnaire was created specifically for the current study, and was administered to parents of cases and controls likewise. Besides the basic sociodemographic information, our questionnaire explores different types of prenatal exposures as well as perinatal complications in participants of the study. The questionnaire was administered during study period and comprized questions regarding presence and quantity of specific exposure.

### DNA isolation and genotyping

Total DNA was isolated from 200 µl of the whole peripheral blood using *QIAamp DNA Blood Mini Kit* (*Qiagen, Chatsworth CA, USA*), in accordance with the manufacturers protocol. Genotyping was performed blinded to the case-control status. Blinded quality control samples were applied for validation of genotyping procedures. Concordance for the blinded samples was 100%. All of the assays included positive and negative controls. All primers used are synthesized and bought from *Metabion International AG* (*Planegg, Germany*)^38^.

### The genotyping of *GSTM1* and *GSTT1*

Multiplex polymerase chain reaction (PCR) method of *Abdel-Rahman et al*.^39^ was done for assessing the presence of amplified PCR products of GSTM1: 215 bp, *GSTT1*: 481 bp, as well as housekeeping gene *CYP1A1*: 312 bp, which was applied as internal control. It is important to emphasize that the assay does not make a distinction between heterozygous or homozygous wild-type genotypes. Therefore, it notes only the presence (at least one allele present, homozygote or heterozygote - *GSTM1 active* and *GSTT1 active* genotype, respectively) or the absence (complete deletion of both alleles, homozygote - *GSTM1 null* and *GSTT1 null* genotype, respectively) of the specific genotype. PCR products were visualized on *Chemidoc (Biorad, Hercules, CA, USA*).

### The genotyping of GSTA1*C69T (rs3957357)

The analysis of the SNP GSTA1*C69T(rs3957357) was performed using PCR-restriction fragment length polymorphism (RFLP) by method of *Ping et al*.^40^. A 400 bp fragment was amplified and subjected to overnight incubation at 37 °C with enzyme *EarI* (*Thermo Fisher Scientific, Waltham, Massachusetts, USA*). Digested products (*GSTA1* CC: 400 bp, *GSTA1* CT: 400 bp + 308 bp + 92 bp and *GSTA1* TT: 308 bp + 92 bp) were visualized on *Chemidoc (Biorad, Hercules, CA, USA*).

### The genotyping of GSTP1*Ile105Val(rs1695)

For assessment of SNP polymorphism *GSTP1 Ile105Val*, TaqMan® SNP Genotyping Assays (*Life Technologies, Applied Biosystems, Carlsbad, CA, USA, assay ID: C__3237198_20*) was performed for amplifying and detecting respective SNP alleles in purified genomic DNA samples, complying to the manufactures’ instructions. DNA concentration and purity were analyzed spectrophotometrically using *GeneQuantpro (Biochrom, Cambridge, England)*. The presence of *GSTP1 Ile/Ile* genotype was defined as *GSTP1-wild* type, whereas the presence of *GSTP1 Ile/Val* or *GSTP1 Val/Val* genotype as *GSTP1 variant* genotype.

### Statistical analysis

Statistical analysis included, besides descriptive statistics, nonparametric and parametric tests depending on the variable type. χ^2^ and t test were used to test possible differences between case and control group on several control variables. The χ^2^ test was also used for the assessment of possible genotype departure from Hardy-Weinberg equilibrium. Three binary logistic regression models were used to test the predictive effects on ASD, with the following sets of predictors: (1) individual genotypes, (2) individual genotypes and genotype-genotype interactions, and (3) individual genotypes, genotype-genotype interactions, maternal smoking status during pregnancy and smoking- genotype interactions. As effect size indicators we used odds ratio (OR, with the 95% confidence interval), percentage of correct classification and Nagelkerke r^2^. The probability level of ≤0.05 was considered statistically significant. For statistical analysis the SPSS 17.0 statistical software package (SPSS Inc, Chicago, IL, USA.) was used.

### Ethical standards

This study has been approved by the ethics committee of the Institute of Mental Health, University Children’s Hospital and Faculty of Medicine, University of Belgrade, Serbia, and has been performed in accordance with the principles of good clinical practice. Prior to participation in this study, parents/caretakers signed the informed consent.

## Results

Baseline characteristics of ASD cases and respective controls are shown in Table 1. There were no differences in sex and age between the case and control group (p = 0.731 and 0.120, respectively). The differences were observed neither in the maternal age (p = 0.465), nor in the maternal education (p = 0.100). Also, the case and the control group didn’t differ in terms of parity and interpregnancy interval (p = 0.548 and 0.296, respectively). There were no differences observed in paternal age and education as well (p = 0.159 and 0.793). Moreover, we found no significant difference in TSDP status regarding mothers of children with ASD and those included in healthy controls group.

**Table 1:** Baseline characteristics of children and their parents in the case and the control group.

Variable	Cases (n = 113)	Controls (n = 114)	t	Χ^2^	P
Child’s Age (years) X ± SD	9.36 ± 5.88	10.62 ± 6.33	−1.562	/	0.120
**Sex n(%)**					
Male Female	92 (81) 21 (19)	95 (83) 19 (17)	/	0.144	0.731
Maternal age (at child’s birth)	28.45 ± 4.79	27.93 ± 5.42	0.731	/	0.465
**Parity**					
*First pregnancy* *Second pregnancy* *Third pregnancy* *After third pregnancy*	45 (46.4) 38 (39.2) 10 (10.3) 4 (4.1)	50 (48.5) 40 (38.8) 12 (11.7) 1 (1)	/	2.118	0.548
Inter-pregnancy interval	3.86 ± 3.12	4.52 ± 3.33	−1.051	/	0.296
**Maternal education**					
*Primary school* *Secondary school* *More than secondary school*	10 (9.9) 43 (42.6) 48 (47.5)	5 (4.9) 58 (56.3) 40 (38.8)	/	4.602	0.100
Paternal age (at child’s birth)	32.93 ± 6.32	31.69 ± 6.12	1.415	/	0.159
**Paternal education**					
*Primary school* *Secondary school* *More than secondary school*	8 (8.1) 57 (57.6) 34 (34.3)	8 (7.8) 64 (62.1) 31 (30.1)	/	0.464	0.793
**Maternal smoking during pregnancy**					
Yes No	30 (30.6) 68 (69.4)	23 (22.3) 80 (77.7)	/	1.774	0.203

The information on parity was obtained from 97 cases and 103 controls, information on mother’s education for 101 cases and 103 controls; on father’s education for 99 cases and 103 controls and on maternal smoking during pregnancy, information was obtained from 98 cases and 103 controls.

### The association of *GST* genotypes with ASD risk

Analyses of Hardy-Weinberg equilibrium have shown that all but one (GSTP1) variants have not deviated from expected distribution. Binary logistic regression was used to test possible prediction of ASD based on individual genotypes. Obtained results showed that *GSTM1 active* genotype decreased the risk for ASD (OR = 0.554, 95%CI: 0.313–0.983, p = 0.044) compared to *GSTM1 null* genotype. It seems that polymorphisms in GSTT1 and GSTP1 genotypes did not contribute to ASD risk. Regarding GSTA1 genotype, carriers of *GSTA1 CC* genotype were at increased risk for ASD (OR = 4.132, 95%CI: 1.219–14.012, p = 0.023) compared to carriers of *GSTA1 CT and GSTA1 TT* genotypes. Percentage of correct classification was 62.5% with Nagelkerke r^2^ = 0.079 (Table 2).

**Table 2:** Individual GST genotypes as predictors of ASD.

Predictor	Wald	Sig.	OR	95% C.I. for OR	
				Lower	Upper
GSTM1	4.071	0.044	0.554	0.313	0.983
GSTT1	0.536	0.464	1.270	0.670	2.405
GSTA1-CC	5.186	0.023	4.132	1.219	14.012
GSTA1-CT	1.862	0.172	2.340	0.690	7.934
GSTP1-llelle	1.566	0.211	0.628	0.304	1.301
GSTP1-ValVal	0.974	0.324	0.597	0.215	1.662
Constant	1.396	0.239	0.460		

GSTM1 and GSTT1 are binary variables (1 active, 0 null), whereas GSTA1 and GSTP1 each have three variants transformed into two dummy variables (1 present, 0 not present). Deletion *GSTM1* and *GSTT1* genotypes were investigated in 112 cases and 108 recruited controls. GSTA1 polymorphism was investigated in 112 cases and 105 controls. GSTP1 Ile105Val polymorphism was analyzed in 111 cases and 108 controls.

After obtaining individual effects, we were also interested in combined effect of any two genotypes (Table 3). We also used binary logistic regression to test possible prediction of ASD based on individual genotypes and all of their interactions. Interestingly, all effects of individual genotypes turned out to be insignificant, while several interactions appeared as significant. Combination of *GSTM1 active* and *GSTT1 active* decreased the risk of ASD (OR = 0.126, 95%CI: 0.029–0.547, p = 0.006), as well as the combination of *GSTT1 active* and *GSTP1 llelle* (OR = 0.170, 95%CI: 0.029–0.992, p = 0.049). On the other hand, increased risk of ASD was noticeable if combination of *GSTM1 active* and *GSTP1 llelle* was present (OR = 11.088, 95%CI: 1.745–70.456, p = 0.011). Other interactions of genotypes did not cross the level of statistical significance. Percentage of correct classification increased a bit in comparison to effects of individual genotypes (65.7% with Nagelkerke r^2^ = 0.202).

**Table 3:** Interactions between GST genotypes as predictors of ASD (controlling for effects of individual genotypes).

Predictor	Wald	Sig.	OR	95% C.I. for OR	
				Lower	Upper
GSTM1	0.691	0.406	3.889	0.158	96.461
GSTT1	0.701	0.402	3.684	0.174	77.967
GSTA1-CC	0.720	0.396	4.521	0.139	147.557
GSTA1-CT	0.333	0.564	2.809	0.084	93.736
GSTP1-llelle	0.435	0.509	3.478	0.086	141.015
GSTP1-ValVal	0.000	1.000	5.228	0.000	.
GSTM1*GSTT1	7.653	0.006	0.126	0.029	0.547
GSTM1*GSTMA1-CC	0.204	0.652	0.484	0.021	11.286
GSTM1*GSTMA1-CT	0.365	0.546	0.381	0.017	8.734
GSTM1*GSTP1-llelle	6.503	0.011	11.088	1.745	70.456
GSTM1*GSTP1-ValVal	0.066	0.798	1.371	0.123	15.289
GSTT1*GSTA1-CC	0.049	0.825	1.411	0.067	29.733
GSTT1*GSTA1-CT	0.042	0.838	1.378	0.064	29.733
GSTT1*GSTP1-llelle	3.877	0.049	0.170	0.029	0.992
GSTT1*GSTP1-ValVal	0.000	0.999	0.000	0.000	.
GSTA1-CC*GSTP1-llelle	0.416	0.519	0.296	0.007	11.956
GSTA1-CC*GSTP1-ValVal	0.000	0.999	3.263E8	0.000	.
GSTA1-CT*GSTP1-llelle	0.436	0.509	0.287	0.007	11.663
GSTA1-CT*GSTP1-ValVal	0.000	0.999	6.062E8	0.000	.
Constant	0.866	0.352	0.195		

GSTM1 and GSTT1 are binary variables (1 active, 0 null), whereas GSTA1 and GSTP1 each have three variants transformed into two dummy variables (1 present, 0 not present). *Indicates genotype-genotype interactions.

We further assessed the effect of smoking during pregnancy in relation to *GST* genotype (Table 4). By binary logistic regression we tested possible prediction of ASD based on individual genotypes, all of their interactions and their interactions with smoking during pregnancy. Same as in previous analysis, all effects of individual genotypes turned out to be insignificant, while same interactions remained significant. Combination of *GSTM1 active* and *GSTT1 active* decreased the risk of ASD (OR = 0.152, 95%CI: 0.029–0.784, p = 0.024), as well as combination of *GSTT1 active* and *GSTP1 llelle* (OR = 0.117, 95%CI: 0.015–0.938, p = 0.043), while combination of *GSTM1 active* and *GSTP1 llelle* increased the risk of ASD (OR = 27.136, 95%CI: 3.424–215.054, p = 0.002). Other interactions of genotypes did not cross the level of statistical significance. The most important finding is that neither effect of smoking during pregnancy per se, neither it’s interaction with any of the genotypes crossed the level of statistical significance. Smoking during pregnancy did not show any effects on the risk of ASD in our study. Percentage of correct classification was similar as in previous analysis which included all effects except smoking during pregnancy (66.3% with Nagelkerke r^2^ = 0.270).

**Table 4:** Smoking during pregnancy and its interactions with GST genotypes as predictors of ASD (controlling for effects of individual genotypes and genotype-genotype interactions).

Predictor	Wald	Sig.	OR	95% C.I. for OR	
				Lower	Upper
GSTM1	0.063	0.802	1.646	0.033	81.028
GSTT1	0.000	0.999	6.745E9	0.000	.
GSTA1-CC	0.000	0.999	5.290E9	0.000	.
GSTA1-CT	0.000	0.999	3.300E9	0.000	.
GSTP1-llelle	0.898	0.343	8.549	0.101	723.627
GSTP1-ValVal	0.000	1.000	0.000	0.000	.
GSTM1-GSTT1	5.063	0.024	0.152	0.029	0.784
GSTM1*GSTMA1-CC	0.081	0.776	0.580	0.014	24.696
GSTM1*GSTMA1-CT	0.104	0.747	0.541	0.013	22.566
GSTM1*GSTP1-llelle	9.768	0.002	27.136	0.182	215.054
GSTM1*GSTP1-ValVal	0.418	0.518	2.312	0.182	29.365
GSTT1*GSTA1-CC	0.000	0.999	0.000	0.000	.
GSTT1*GSTA1-CT	0.000	0.999	0.000	0.000	.
GSTT1*GSTP1-llelle	4.082	0.043	0.117	0.015	0.938
GSTT1*GSTP1-ValVal	0.000	1.000	0.000	0.000	.
GSTA1-CC*GSTP1-llelle	0.904	0.342	0.126	0.002	9.013
GSTA1-CC*GSTP1-ValVal	0.000	0.999	1,746E27	0.000	.
GSTA1-CT*GSTP1-llelle	1.684	0.194	0.058	0.001	4.298
GSTA1-CT*GSTP1-ValVal	0.000	0.999	1.746E27	0.000	.
Smoker	0.000	0.999	3.284E18	0.000	.
GSTM1*smoker	0.623	0.430	2.015	0.354	11.481
GSTT1*smoker	0.232	0.630	0.659	0.120	3.603
GSTA1-CC*smoker	0.000	0.999	0.000	0.000	.
GSTA1-CT*smoker	0.000	0.999	0.000	0.000	.
GSTP1-llelle*smoker	0.012	0.911	1.143	0.109	12.038
GSTP1-ValVal*smoker	0.262	0.609	2.177	0.111	42.861
Constant	0.000	0.999	0.000		

GSTM1 and GSTT1 are binary variables (1 active, 0 null), whereas GSTA1 and GSTP1 each have three variants transformed into two dummy variables (1 present, 0 not present).

*Indicates genotype-genotype interactions and interactions between smoking status during pregnancy and genotype.

## Discussion

The present study investigated polymorphic expression in four classes of glutathione S-transferases (GSTA1, GSTM1, GSTT1 and GSTP1) in individuals with ASD, as well as their possible gene-gene and gene-environment interaction underlying this disorder. The results of this study showed that the *GSTM1 null* and *GSTA1 CC* genotypes were significantly more frequent in patients with ASD. To the best of our knowledge, these are the first results presented in the literature focused on clarifying the association of individual *GSTA1* polymorphism and ASD. On the other hand, *GSTM1 active* genotype seems to be protective in terms of ASD development. Similarly, combined *GSTM1 active* and *GSTT1 active* genotype, as well as, combined *GSTT1 active* and *GSTP1 IleIle* genotype also decrease ASD risk. Another interesting finding is the observed effect of *GSTM1 active* and *GSTP1 IleIle* genotypes which were significantly associated with susceptibility to ASD.

Over the years, many studies have attempted to elucidate triggering genetic factors in the development of ASD^4^, however, so far only several studies evaluated the possible association between GST polymorphisms, as independent factor (or in conjunction with environmental factors), and susceptibility to ASD^10,11,16,17^. Cytosolic GST family catalyzes the conjugation of electrophilic compounds, including products of oxidative stress, with GSH^9^. Polymorphisms within GST classes result in complete lack or altering of enzyme activity, hence altering both the capacity for detoxification of different endogenous and exogenous compound, including oxidants, and in that way contributing to development of various neurological and mental disorders, along with ASD^9,17,41–44^.

The emphasis regarding GST polymorphisms in autism spectrum disorders has been put on *GSTM1* and *GSTT1* deletion polymorphisms, which, in carriers of *GSTM1 null* or *GSTT1 null* genotype, affect cell’s ability to metabolize toxins due to complete lack of active enzyme^9,33^. Several studies found increased ORs for ASD in carriers of *GSTM1 null* genotype alone or in combination with other genetic factors^16,18^. Our findings on increased *GSTM1 null* genotype frequency among cases in comparison to controls, together with protective role of GSTM1 active genotype in ASD development are in agreement with a study by Buyske *et al*.^18^, while the study by Rahbar *et al*.^17^, didn’t show this result. Although this significance was not shown when genotype and genotype interactions and TSDP were added into the model, *GSTM1 active* genotype showed significant interaction with *GSTT1 active* genotype, significantly decreasing the risk for ASD, as well as with *GSTP1 IleIle* genotype, with the opposite effect.

The other GST deletion polymorphism, as mentioned, results in *GSTT1 null* genotype, which might be considered as either risk-associated or protective in certain disorders,(due to its role in detoxification, but also in bioactivation). Namely, it is well established that GSTT1 enzyme is involved in bioactivation, rather than detoxification of several bifunctional alkylating agents, present in environmental pollution and certain occupational hazards^45^. So far, the possible association between *GSTT1* genotype and ASD was investigated only in one study in which no association between individual *GSTT1* genotype and risk of ASD development was observed^17^ and our results are in agreement with this finding. However, we found that the combination of *GSTT1 active* genotype might act protectively in combination with specific GST genotypes. Our results show that the combinations of *GSTT1 active* and *GSTM1 active* genotype, as well *GSTT1 active* with *GSTP1 IleIle* genotypes, decrease the risk for ASD. Similar to our results, the combined *GSTT1 null* and *GSTP1 IleIle* or *IleVal* genotype was recognized as significant in terms of risk of ASD in Jamaican population^17^.

Alpha class of GSTs, which is expressed in most tissues, including brain^45^, is specific for its substrate promiscuity as a consequence of protein flexibility and dynamics in the enzymes active site^46^. It also possesses peroxidase activity towards organic hydroperoxides and might be involved in regulation of cellular redox homeostasis, and therefore it is interesting that to date, it has not been analyzed in ASD. Moreover, Iorio *et al*.^47^ provided evidence supporting the notion that *GSTA1* may play an important role during pregnancy, since previous studies indicated that *GSTA1* polymorphism is associated with different pregnancy-related conditions^48–50^. Polymorphism of GSTA1 is represented by three linked single nucleotide polymorphisms (SNPs), resulting in differential expression with lower transcriptional activation of the variant GSTA1*B (T) than the common GSTA1*A (C) allele^33^. Results of this study have shown that carriers of *GSTA1 CC* genotype, with the highest expression of GSTA1 enzyme, are in increased risk of developing ASD when compared to individuals with *GSTA1 CT* and *TT* genotype. The possible explanation for the observed associations might be in the fact that, similarly to GST theta class, alpha class also participates in bioactivation of several drugs and certain neurotoxic compounds^51^. This significance was lost in further analysis, both for individual and combined effect of GSTA1 genotype status.

Another important SNP in glutathione S-transferase superfamily is GSTP1 polymorphism, which has been suggested as contributing factor in ASD development years ago. As mentioned, the risk of ASD was observed in offspring of mothers with *GSTP1 IleVal* genotype^10^, as well as, in carriers of *GSTP1 wild type* genotype depending on blood manganese concentration or exposure to aluminum^11,15^.

Results of our study failed to associate individual *GSTP1* genotype with ASD, while it seems that in combination with other GST polymorphisms *GSTP1 IleIle* genotype contributes differentially to the effect on ASD. The lack of association of individual GSTP1 genotype with ASD has also been shown in the study by Rahbar *et al*.^17^. Still, the interaction of *GSTP1 IleVal* with *GST1 null* genotype was shown to be significant in the Jamaican sample^17^. Also, it was shown that *GSTP IleIle* genotype interacts with blood manganese levels – it was proven that this genotype increased the risk of ASD significantly, in children with high manganese blood levels^11^. Our results show that *GSTP1 IleIle* genotype was significant in interaction - with *GSTT1 active* decreasing the risk of ASD, and with *GSTM1 active* genotype increasing the risk. The role of GSTP1 genotypes has been shown in several complex disorders, showing complex interactions^17^. Further studies with emphasis of GSTP1 genotypes and other genetic and environmental factors could elucidate its role in complex disorders such as ASD.

We further tried to explore whether there is any gene-environment interaction between different *GST* gene variants and exposure to cigarette smoke metabolites. This possible gene-environment interaction seems to be biologically plausible, since it has been shown that *GSTP1 Val* allele is more catalytically efficient towards benzo-diol-epoxides found in tobacco smoke^52^. Also, glutathione S-transferases are involved in detoxification of both free radicals and reactive polycyclic aromatic hydrocarbon metabolites, all present in cigarette smoke^33^. Namely, due to the fact that GST enzymes belonging to various classes have different, but sometimes overlapping, substrate specificities, their contribution in tobacco smoke metabolism and cellular antioxidant defense must be taken into account^9,32^. The surprising result was that neither the effect of smoking during pregnancy per se, nor it’s interaction with any of the genotypes showed any effects on the risk of ASD. A recent meta-analysis showed that maternal smoking during pregnancy is not associated with ASD risk in offspring^30^, which is in line with our finding. Although the possible interaction between TSDP and GST genetic polymorphisms might explain the inconsistency in results of studies on association of TSDP and risk of ASD^27,30^, our study has not confirmed the hypothesis. To our knowledge, this is the first study to examine this gene-environment interaction in ASD.

Our study has strengths and limitations. The strength of our study is the fact that, to our knowledge, this is the first study that examined GSTA1 genotypes in risk for ASD. Also, this is one of the rare studies that examined not only individual, but also combined effects of specific GST genotypes on risk for this group of disorders.

Certain limitations might also be considered in our study. The case–control design was used for estimating of associations between individual and combined GST genotypes and increased risk for ASD, and therefore the selection bias might influence the results. Furthermore, our control group was hospital-based and relatively small; therefore, the use of population controls might have been more appropriate. In this line, the possible effect of ethnicity could not be evaluated since the study subjects were Caucasian only. Therefore, further genotyping, using a larger sample size, is needed to better understand association between GST gene variants, relevant environmental exposures and increased risk for ASD. It is of note that in case of the GSTP1 variant we have observed deviation from Hardy-Weinberg equilibrium. Although this deviation could be due to relatively small sample size, our results for GSTP1 must be taken with caution and future studies will be needed to comprehensively assess the effect of this variant.

Taken together, polymorphic expression of glutathione S-transferases might influence individual susceptibility to autism spectrum disorders, especially taking into account different oxidative stress-specific gene-environment interaction. If it could be possible to identify persons at higher risk of ASD based on specific oxidative stress genotype, possible preventive actions might be taken.

Still, the presence of different GST gene variants needs to be analyzed in conjunction with other genetic and environmental risk factors. That way, the fact that a certain prenatal factor has not been firmly associated with the increased risk of ASD in the general population does not exclude the possibility that there is a sensitive subpopulation (genotype wise).

## Data Availability

All data generated or analyzed during this study are included in this published article.
